# eIF4A inhibition prevents the onset of cytokine-induced muscle wasting by blocking the STAT3 and iNOS pathways

**DOI:** 10.1038/s41598-018-26625-9

**Published:** 2018-05-30

**Authors:** Zvi Cramer, Jason Sadek, Gabriela Galicia Vazquez, Sergio Di Marco, Arnim Pause, Jerry Pelletier, Imed-Eddine Gallouzi

**Affiliations:** 1 McGill University, Department of Biochemistry, Rosalind and Morris Goodman Cancer Centre, Montreal, Quebec, Canada; 2 Hamad Bin Khalifa University (HBKU), College of Science and Engineering, Life Sciences Division, Education City, Doha, PB 5825 Qatar

## Abstract

Cachexia is a deadly muscle wasting syndrome that arises under conditions linked to chronic inflammation, such as cancer. Cytokines, including interferon γ (IFNγ), tumor necrosis factor α (TNFα) and interleukin-6 (IL-6), and their downstream effectors such as Signal Transducer and Activator of Transcription 3 (STAT3), have been shown to play a prominent role in muscle wasting. Previously, we demonstrated that Pateamine A (PatA), a compound that targets eukaryotic initiation factor 4A (eIF4A), could prevent muscle wasting by modulating the translation of the inducible Nitric Oxide Synthase (iNOS) mRNA. Here we show that hippuristanol, a compound that impedes eIF4A in a manner distinct from PatA, similarly inhibits the iNOS/NO pathway and cytokine-induced muscle wasting. Furthermore, we show that hippuristanol perturbs the activation of the STAT3 pathway and expression of STAT3-gene targets such as IL-6. The decreased activation of STAT3, which resulted from a decrease in STAT3 protein expression, was due to the inhibition of STAT3 translation as there were no changes in STAT3 mRNA levels. These effects are likely dependent on the inhibition of eIF4A activity since we observed similar results using PatA. Our results identify the inhibition of eIF4A-responsive transcripts, such as STAT3, as a viable approach to alleviate cachexia.

## Introduction

Cachexia, a multi-factorial disease characterized by acute muscle wasting and weight loss, accompanies various inflammatory diseases such as cancer, sepsis and AIDS^1,2^. The abnormal catabolic state found in cachectic patients arises from a combination of complex metabolic changes and dysregulation of certain humoral factors^3–5^. Cachexia is the primary cause of ~22% of cancer-related deaths^6^ and has been known for decades as being a major influence on mortality rate in cancer patients. In spite of the relevance of this syndrome as a contributor to cancer-related deaths, there are no widely employed therapeutics that effectively alleviate this disease^7^.

Despite the convoluted etiology of cachexia, some important mediators of its underlying pathophysiology have been identified. Accumulating evidence depicts certain pro-inflammatory cytokines and their downstream effectors as playing pivotal roles in the onset of cancer cachexia^7,8^. For example, the concurrent signaling of interferon γ (IFNγ) and tumor necrosis factor α (TNFα) (IT) can synergistically elicit muscle wasting by stimulating the activity of transcription factors including STAT3 and the heterodimeric NF-κB^9–12^. NF-κB signaling in skeletal muscle upregulates the muscle-specific E3 ligase MURF-1 and induces a loss of proteins integral for muscle fiber formation and maintenance, such as MyoD and Myogenin^7,9,11,13^. Moreover, we have shown that NF-κB can also mediate muscle wasting by collaborating with STAT3 to markedly increase the transcription of inducible nitric oxide synthase (iNOS), an enzyme that catalyzes the conversion of _L_-arginine to citrulline resulting in the release of nitric oxide (NO)^7,9,10,12^. Systemic interleukin-6 (IL-6) signaling is also crucial in inducing muscle wasting and has been shown to be involved in the pathophysiology of at least some models of cancer cachexia^6,14–17^. Chronic IL-6 exposure has been directly linked to the aberrant activation of autophagic and ubiquitin-proteasomal degradation systems in the muscle^17^. Furthermore, many studies have shown the importance of STAT3 in the muscle wasting process in a variety of IL-6-dependent models of cancer cachexia. These observations demonstrate that STAT3 is essential in cachexia driven by a multitude of cytokines including IFNγ, TNFα and IL-6^18–22^. Attempts at interfering with cytokine signaling to impede cachexia progression have included the use of antibodies targeting TNFα or IL-6, however the success of these therapeutic approaches was very limited^23,24^. The disappointing outcomes in these trials could be due to the involvement of multiple distinct pathways, the cooperation of which is required for cachexia onset or due to redundancy in the downstream effectors of TNFα and IL-6, such as STAT3^12^. In light of these results, therapies that can disrupt multiple pathways or target redundant factors downstream of these humoral factors may be a more fruitful approach to combatting cachexia.

Disrupting the initiation of eukaryotic mRNA translation, including the rate-limiting recruitment of the 40S ribosome via the eIF4F complex, has been shown to have anti-immunogenic, anti-oncogenic and anti-cachectic effects^25–27^. Compounds such as silvestrol, pateamine A (PatA) and hippuristanol (Hipp) mediate these effects by inhibiting the function of eIF4A, a RNA helicase component of eIF4F that unwinds complex secondary structures in mRNAs^28^. These compounds are believed to act in this manner by perturbing the translation of specific set of mRNAs containing complex secondary structures in their 5′ untranslated region (UTR) that hinder ribosomal recruitment^27–31^. Hipp is an allosteric inhibitor that prevents eIF4A binding to RNA^32^ whereas PatA and silvestrol deplete eIF4A from the eIF4F complex by causing eIF4A to clamp onto RNA^33,34^ thus disrupting the interplay between eIF4A and dependent transcripts^35^. In the past decade, these and other compounds that target the eIF4F complex have received considerable attention, with several in preclinical development^25^.

The anti-inflammatory effects of compounds that alter eIF4A function prompted us to investigate their impact in cancer cachexia. Previously, we reported that low doses of PatA prevents cytokine-induced muscle wasting both *in vitro* and *in vivo* in a C26-adenocarcinoma tumour induced mouse model of muscle wasting^27^. Without affecting general translation, we found that this low dose of PatA selectively disrupts the translation of iNOS mRNA by increasing its affinity to eIF4A, suggesting that targeting iNOS via eIF4A may be an efficacious clinical strategy for alleviating cachexia. Although hindering iNOS translation likely contributes to the efficacy of PatA, the observation that impairing eIF4A is more efficacious than the iNOS inhibitor aminoguanidine (AMG) in preventing cachexia in *vivo*^27^, indicates that the expression of other eIF4A-dependent transcripts may also be altered by PatA. The fact that PatA action is irreversible and could be toxic^33,34^ put into question whether PatA could be a viable treatment option for cancer cachexia in humans. Herein, we tested whether other eIF4A-inhibiting compounds can, similarly to PatA, prevent cytokine induced-muscle wasting as well as whether this strategy impacts other pro-cachectic factors besides iNOS. We report that Hipp, which differs with PatA in its mode of inhibiting eIF4A function, prevented the onset of cytokine-induced muscle wasting due, in part, to curtailing the induction of the iNOS/NO pathway. Furthermore, we show for the first time, that compounds targeting eIF4A function modulate the activation of the STAT3 pathway. These drugs decreased STAT3 protein levels in IFNγ/TNFα -treated myotubes without a concurrent effect on the STAT3 mRNA suggesting that eIF4A regulates the translation of STAT3 mRNA under these conditions. Our study therefore indicates that targeting eIF4A function is a robust means of alleviating muscle wasting due to its ability to regulate the translation of specific pro-inflammatory mRNAs, such as iNOS and STAT3, which are involved in the activation of multiple downstream pro-cachectic pathways.

## Results

### The eIF4A allosteric inhibitor Hippuristanol recapitulates the actions of Pateamine A on cytokine-induced muscle wasting and the iNOS/NO pathway

**Figure 1:**
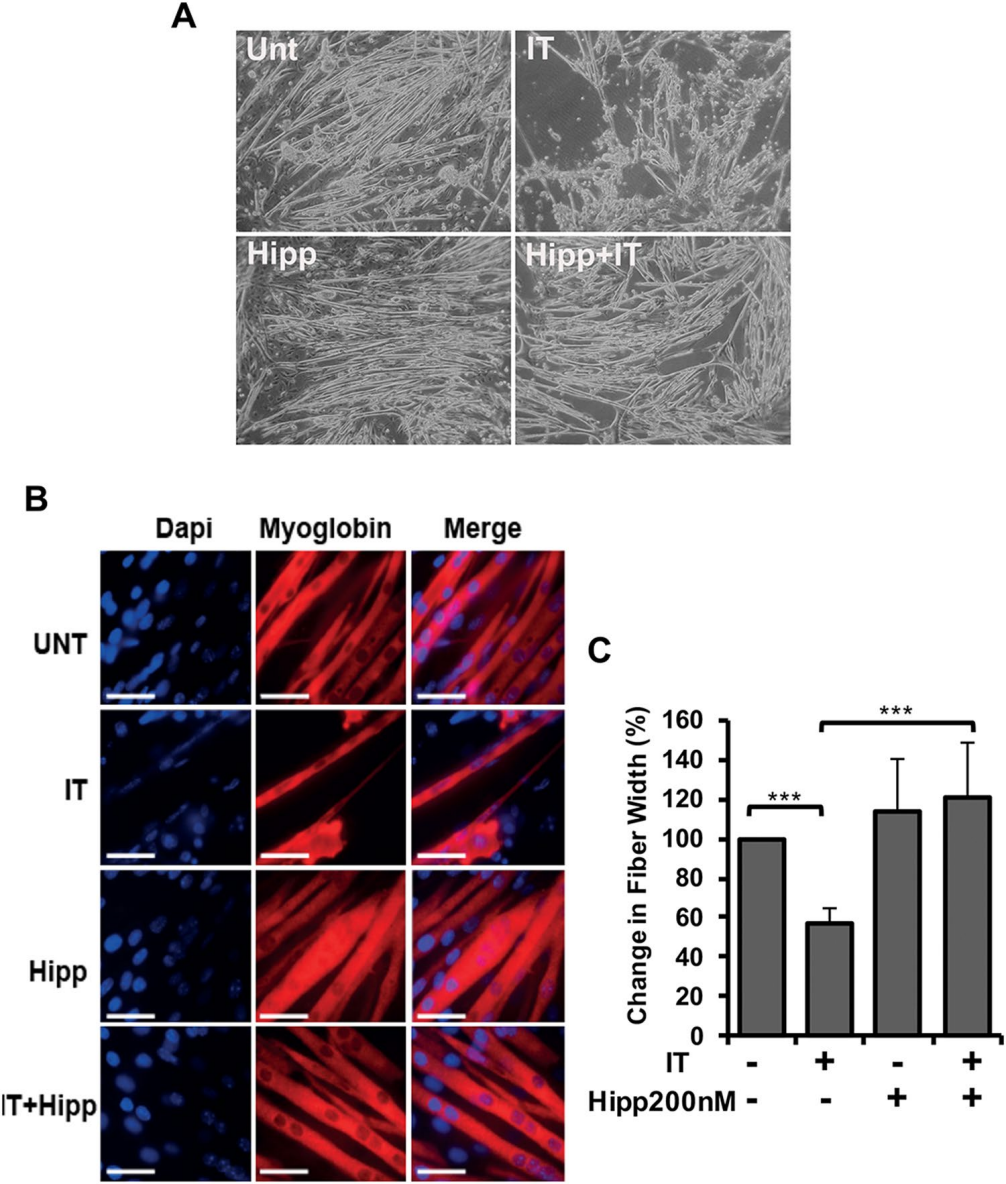
Hippuristanol (Hipp), an eIF4A inhibitor, prevents IT-induced muscle wasting. (**A**) Phase-contrast image of myotubes treated with or without IFNγ (100U mL-1) and TNFα (20 ng/mL) (IT) for 72 hours in the presence or absence Hipp (200 nM). (**B**) Myotubes were treated as described in (**A**). Myotubes integrity was visualized by immunofluorescence using an anti-myoglobin antibody. (**C**) Fiber widths from the immunofluorescence experiment in (**B**) were measured and plotted as the percentage relative to untreated myotubes ± s.e.m ***P < 0.001 (n = 3).

**Figure 2:**
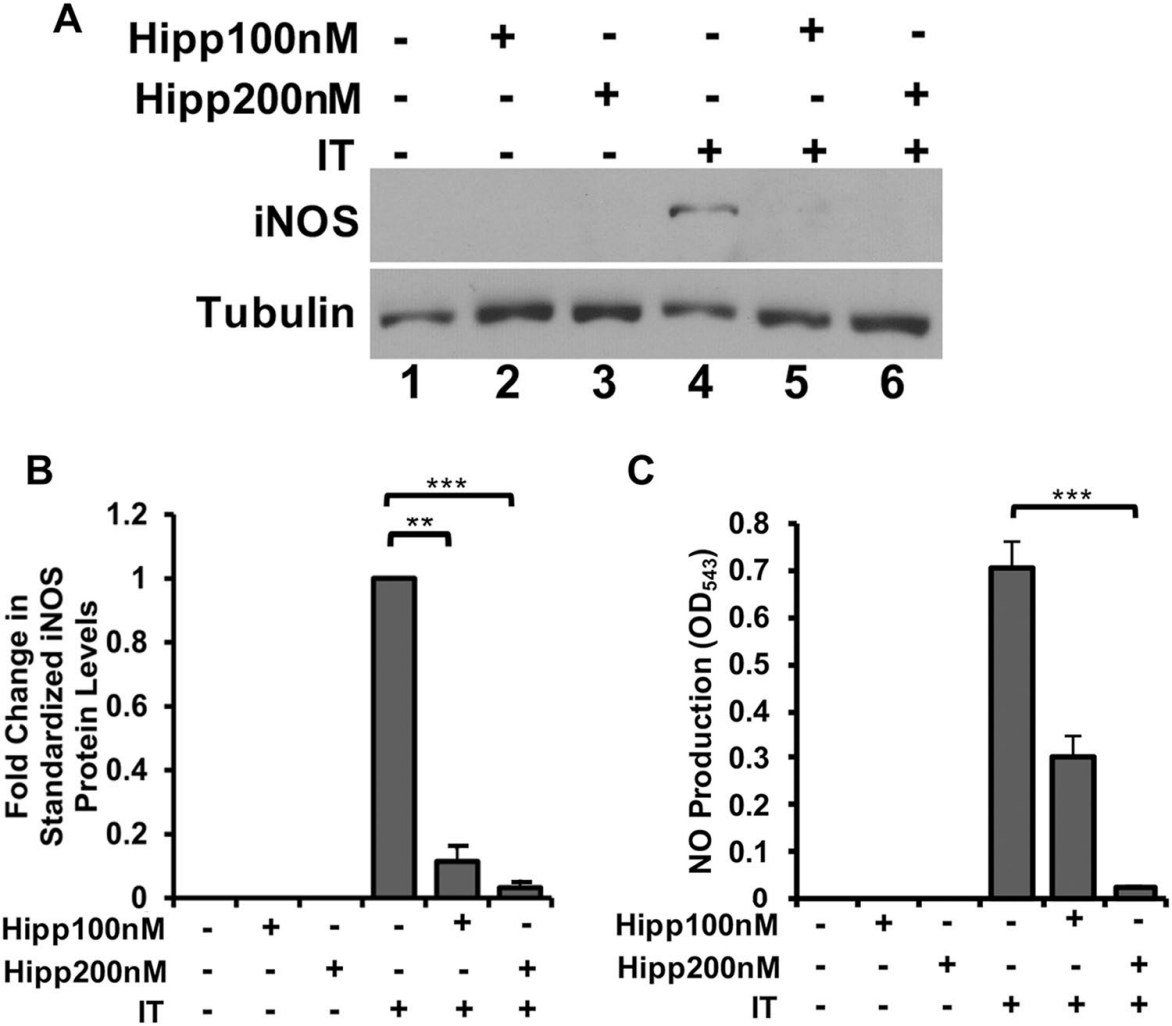
Hippuristanol (Hipp) prevents IT-mediated activation of the pro-cachectic iNOS/NO pathway. (**A**) Western blot analysis of iNOS protein levels in myotubes treated with or without IT in the presence or absence of Hipp (100 and 200 nM) was performed using an anti-iNOS antibody. Tubulin was included as a loading control. (**B**) The aforementioned Western blot for iNOS was quantified and standardized to Tubulin levels. Data are shown relative to IT-induced iNOS protein levels and plotted ± s.e.m **P < 0.01, ***P < 0.001 Student’s T-test (n = 3). (**C**) NO production in myotubes treated as described in (**A**) was determined using the Griess assay and plotted ± s.e.m ***P < 0.001 Student’s T-test (n = 4).

The unfavorable toxicity of high doses of PatA led us to investigate the effect of Hipp, a non-toxic eIF4A-targeting compound^36,37^, on our *in vitro* models of cachexia-induced muscle wasting. A popular *in vitro* model of cachexia involves the treatment of C2C12 myotubes with IFNγ and TNFα (IT) for 72 h, recapitulating many facets of the cachectic muscle^9,11,38^. In order to assess the impact of Hipp on cytokine-induced muscle wasting, we measured the fiber widths of myotubes treated with or without IT and/or Hipp for 72 hours^27^. We found that, like PatA, the presence of Hipp significantly prevented myotube wasting driven by IT (Fig. 1A–C). Next, we tested the effect of different doses of Hipp (100 and 200 nM) on iNOS expression, one of the principal effectors of IT signaling that is decreased by PatA. Indeed, both doses of Hipp significantly reduced iNOS protein levels (Fig. 2A,B) and NO secretion (Fig. 2C) as measured by Western blot and Griess reagent, respectively. Together, our findings indicate that Hipp can mirror the actions of PatA in cytokine-induced muscle wasting and iNOS expression in wasting muscle fibers. Thus, our results support the hypothesis that perturbing eIF4A function underlies PatA-mediated inhibition of iNOS protein expression and muscle wasting.

### Hippuristanol reduces cytokine-induced activation of the STAT3 pathway

**Figure 3:**
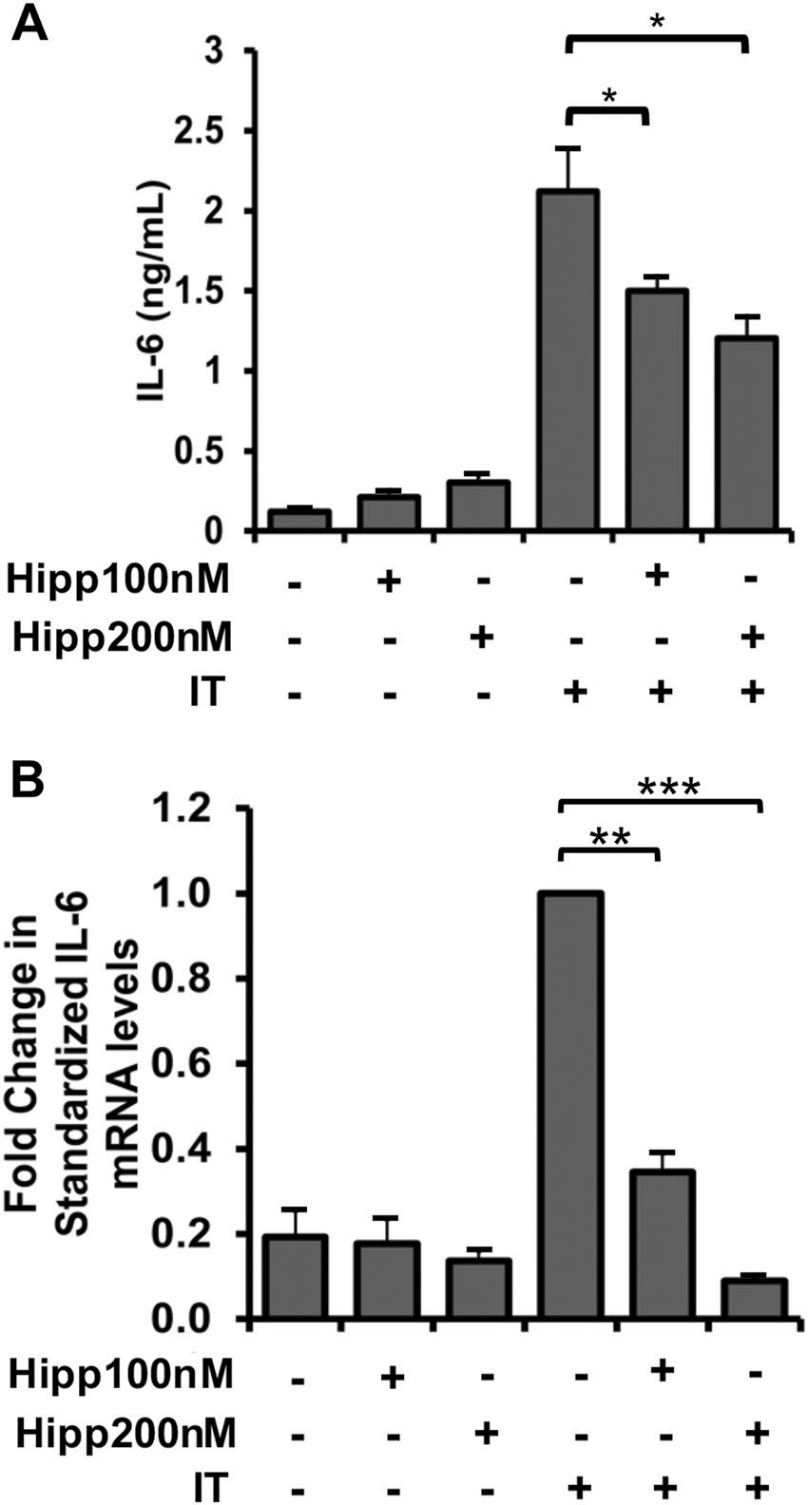
Hippuristanol (Hipp) decreases IL-6 mRNA expression and secretion in IT-treated myotubes. (**A**) ELISA was performed to detect IL-6 levels in the supernatants from myotubes treated with or without IT in the presence or absence of Hipp (100 nM and 200 nM) and plotted ± s.e.m *p < 0.05 Student’s T-test (n = 3). (**B**) RT-PCR was performed using RNA extracted from myotubes treated as described in (A) and the resulting cDNA was quantified using qPCR with primers against IL-6. Values are standardized to the RPL32 housekeeping gene, normalized relative to IT-treated levels and plotted ± s.e.m **p < 0.01, ***p < 0.001 Student’s T-test (n = 3).

Although the reduction of iNOS protein in the presence of Hipp and PatA likely contributes to the effectiveness of these compounds against cytokine-induced muscle wasting, preventing eIF4A-dependent translation likely interferes with the expression of other pro-cachectic genes. Indeed, we found that targeting eIF4A is more effective than the iNOS inhibitor AMG in preventing cachexia in our *in vivo* C26-adenocarcinoma tumour-induced model of muscle wasting^27^. As mentioned above, IL-6 is an important mediator in the onset of muscle wasting in numerous cachexia murine models. Moreover, IL-6 secretion is significantly elevated in myotubes after 24 h of IT exposure^12^. Therefore, we investigated the impact of Hipp on IL-6 mRNA expression and protein secretion. We found that Hipp significantly decreased IL-6 secretion in IT-treated myotubes (Fig. 3A). Additionally, the induction of IL-6 mRNA by IT was markedly impaired in the presence of Hipp (Fig. 3B). While these findings indicate that Hipp decreases IL-6 expression and secretion, the effect on IL-6 mRNA raises the possibility that this inhibitory action likely occurs through an indirect mechanism.

**Figure 4:**
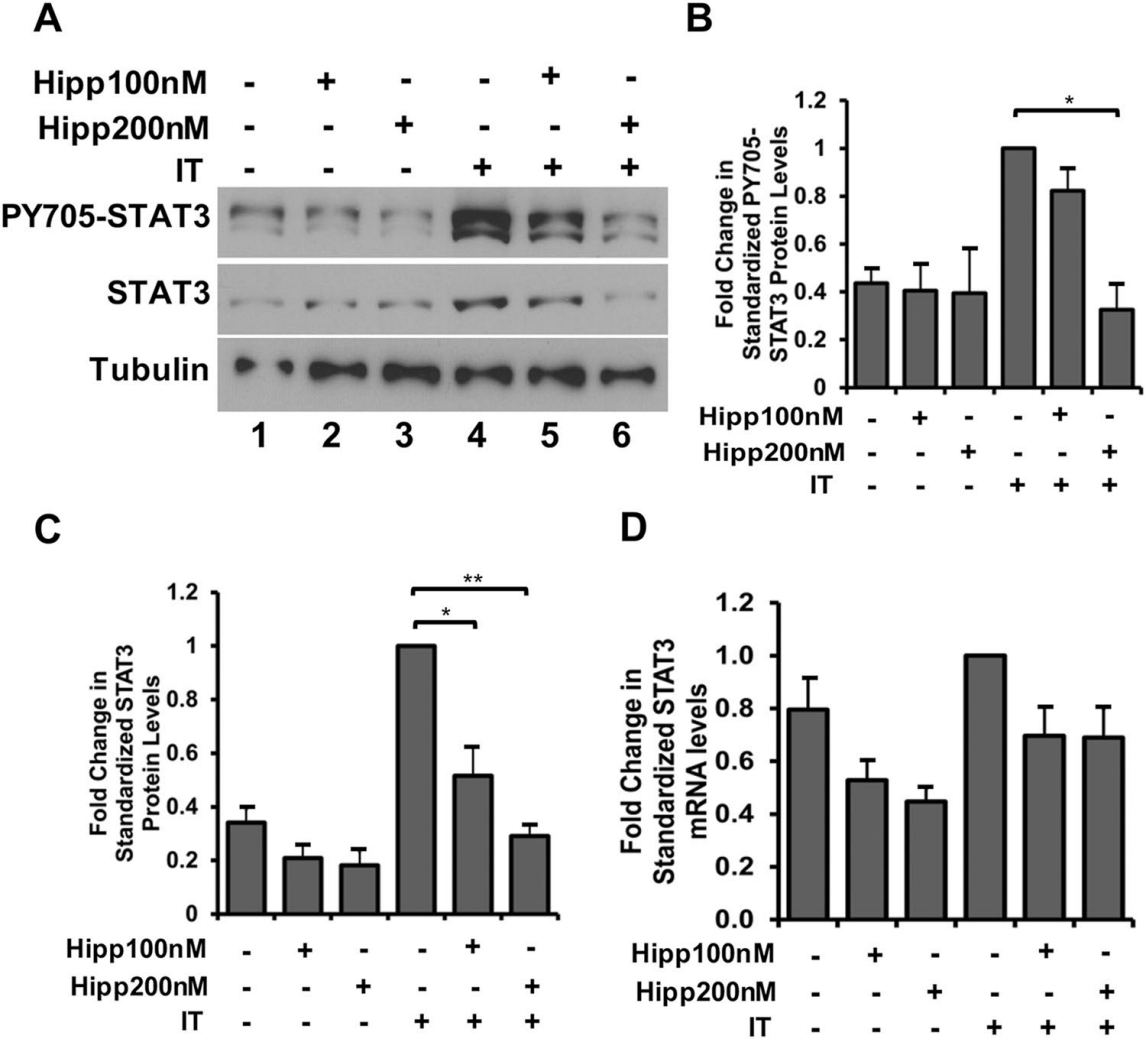
Hippuristanol (Hipp) inhibits STAT3 protein, but not STAT3 mRNA levels, in IT-treated myotubes. (**A**) Western Blot analysis of myotubes treated with or without IT in the presence or absence of Hipp using antibodies against phosphotyrosine^705^ STAT3, STAT3 and Tubulin (loading control). (**B**) phosphotyrosine^705^ STAT3 and (**C**) STAT3 levels shown in the Western blots in (**A**) were quantified, standardized to Tubulin and normalized relative to IT-treated myotubes. Values were plotted ± s.e.m *p < 0.05, **p < 0.01 Student’s T-test (n = 3). (**D**) RT-qPCR analysis on RNA derived from myotubes treated as described in (**A**) using primers against STAT3, standardized to the housekeeping gene RPL32 and normalized relative to IT-treated myotubes. Values were plotted ± the s.e.m. N.S. Student’s T-Test (n = 3).

To account for the change in IL-6 mRNA and protein levels driven by Hipp, we next looked at the activation of pro-cachectic transcription factors that are known to induce IL-6 expression. Indeed, a recent global ribosomal footprinting study has revealed that super enhancer-associated transcription factors are sensitive to eIF4A perturbation^30^. IL-6 is a well-known transcriptional target of STAT3, a super enhancer-associated transcription factor that is essential to the onset of muscle wasting accompanying chronic exposure to both IT and IL-6^12,19,39–41^. Therefore, we analyzed the impact of Hipp on STAT3 activation and protein levels in IT-treated myotubes. We observed that the expression of STAT3 protein and consequently, the induction of its transcriptionally active phosphotyrosine^705^ isoform, were both significantly decreased in the presence of Hipp (Fig. 4A–C). Furthermore, RT-qPCR experiments revealed that STAT3 mRNA levels in IT-treated myotubes were not significantly altered by the addition of Hipp (Fig. 4D). Thus, these results suggest that Hipp prevents activation of the STAT3 pathway by depleting STAT3 protein, likely by directly regulating its translation. Taken together, our data indicates that Hipp may indirectly block IL-6 secretion by inhibiting the translation of STAT3, a transcription factor essential in its expression.

### Pateamine A decreases STAT3 protein levels and IL-6 secretion in cytokine-treated myotubes

**Figure 5:**
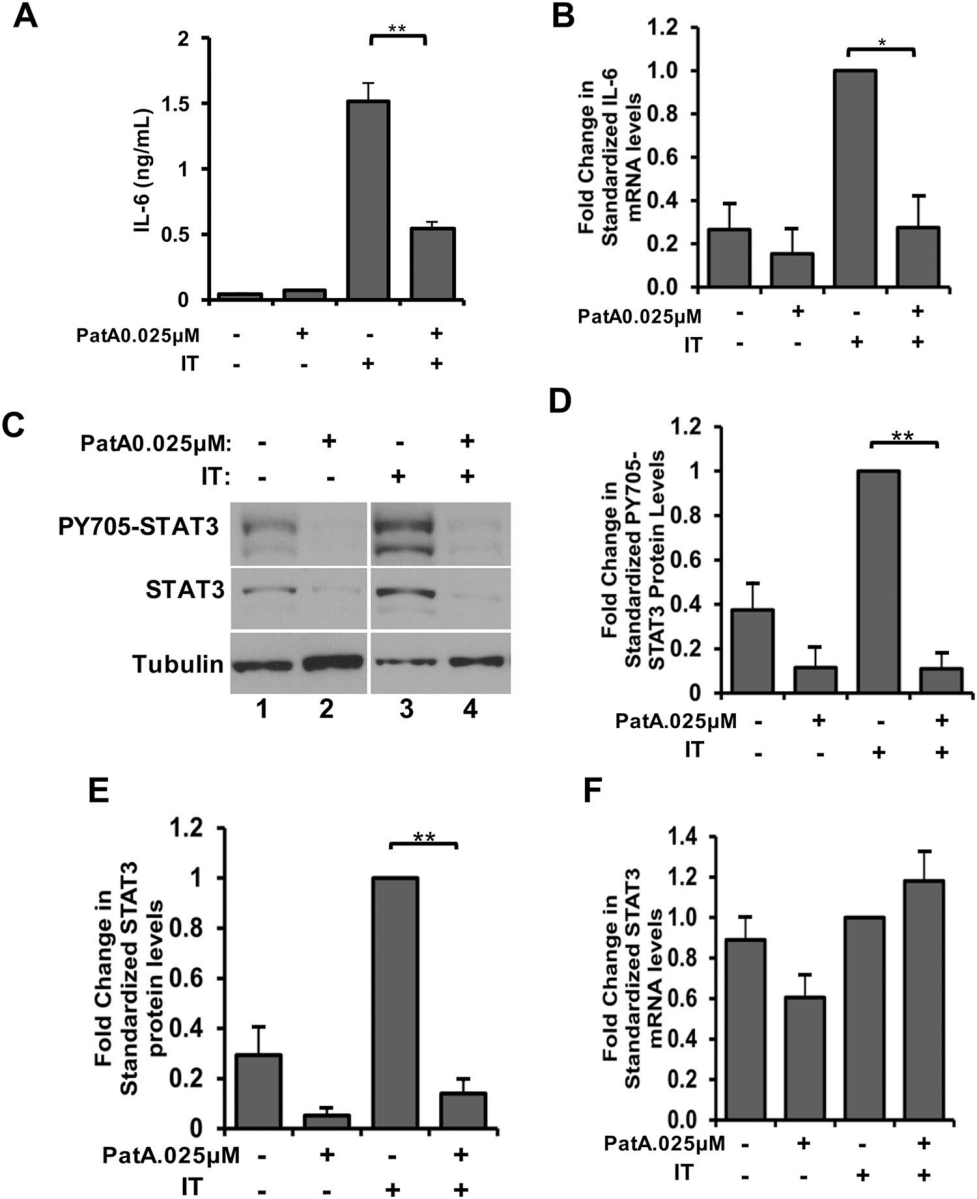
Pateamine A recapitulates the impact of Hippuristanol on IL-6 secretion and STAT3 protein levels. (**A**) IL-6 levels in the supernatant of myotubes treated with or without IT in the presence or absence of PatA (0.025 µM) were determined by ELISA. Values were plotted ± the s.e.m. **p < 0.01 Student’s T-Test (n = 3). (**B**) RT-PCR was performed on RNA extracted from myotubes treated with or without IT in the presence or absence of PatA. cDNA was quantified using qPCR with primers against IL-6. Values were standardized to the RPL32 housekeeping gene, normalized relative to IT-treated levels and plotted ± s.e.m *p < 0.05 Student’s T-test (n = 3). (**C**) Western blot analysis of phosphotyrosine^705^ STAT3 and STAT3 protein levels in myotubes treated as described in (**A**). Tubulin is provided as loading control. (**D**) Phosphotyrosine^705^ STAT3 and (**E**) STAT3 levels shown in the Western blots in (**C**) were quantified, standardized to tubulin and normalized relative to IT-treated myotubes. Values were plotted ± s.e.m **p < 0.01 Student’s T-test (n = 3). (**F**) RT-qPCR analysis on RNA derived from myotubes treated with or without IT in the presence or absence of PatA using primers against STAT3. STAT3 mRNA levels were standardized to the housekeeping gene RPL32 and normalized relative to IT-treated myotubes. Values were plotted ± the s.e.m. N.S. Student’s T-Test (n = 3).

PatA and Hipp similarly impair cytokine-induced muscle wasting and iNOS protein levels, presumably due to the perturbance of eIF4A function. We therefore investigated whether PatA could mirror the impact of Hipp on IL-6 as well as STAT3 to ensure this process is also driven by an eIF4A-dependent mechanism. We observed that, like Hipp, the presence of PatA in myotubes prevented the elevation of IL-6 secretion induced by IT (Fig. 5A). Moreover, we found that PatA significantly decreased IL-6 mRNA levels (Fig. 5B). In addition, similarly to Hipp, PatA significantly decreased STAT3 and phosphotyrosine^705^ STAT3 protein levels (Fig. 5C–E). Nevertheless, STAT3 mRNA abundance was not affected by PatA (Fig. 5F), suggesting that PatA also inhibits STAT3 translation. Thus, PatA recapitulates the changes in IL-6 and STAT3 evoked by Hipp.

In order to further confirm that the effect of PatA and Hipp on STAT3 mRNA translation is dependent on eIF4A, we next investigated the impact of Silvestrol, a compound belonging to a separate class of eIF4A inhibitors termed rocaglamides, on iNOS and STAT3 expression^29,42^. We found that, as with Hipp and PatA, previously established dosages^37^ of Silvestrol significantly decreased NO secretion as well as iNOS protein levels in myotubes treated with IT (Supp. Figure 1A–C). Moreover, Silvestrol significantly depleted STAT3 protein levels without affecting STAT3 mRNA steady state levels (Supp. Figure 1B,D,E). Our results therefore indicate that targeting eIF4A reduces the abundance of multiple proteins, including iNOS, STAT3 and IL-6, which are essential in the onset of cachexia. The obstruction of iNOS and STAT3 may ultimately underlie the effectiveness of these eIF4A-targeting drugs in preventing cachexia-induced muscle wasting.

## Discussion

Although cachexia has been appreciated as a prevalent cause of mortality in the late stages of many chronic inflammatory diseases, there is no treatment regimen that reverses this syndrome. We previously showed that low doses of PatA, a compound that perturbs eIF4A-dependent translation, could prevent the onset of muscle wasting in multiple murine models of cachexia, in part by disrupting iNOS translation. Here, we aimed to determine whether other compounds that act on eIF4A could prevent cytokine-induced muscle wasting as well as identify other pro-cachectic transcripts that are also sensitive to changes in eIF4A function. We demonstrate that Hipp, a natural product that disrupts eIF4A activity differently than PatA, could recapitulate the effects of PatA by preventing the onset of cytokine-induced muscle wasting and by repressing iNOS protein levels. Furthermore, we show that both Hipp and PatA block IL-6 secretion evoked by IT as well as deplete IL-6 mRNA. To potentially account for the decline of IL-6 mRNA levels, we show that STAT3 and phosphotyrosine^705^ STAT protein levels are decreased in the presence of Hipp and PatA as well as another eIF4A inhibitor, Silvestrol, a rocaglamide derivative. Taken together, our results indicate that targeting the activity of eIF4A prevents cytokine-induced muscle wasting by inhibiting the translation of eIF4A-mRNA targets, consequently leading to reduced expression of pro-cachetic factors such as STAT3 and iNOS that are involved in pathways essential in engendering the wasting process.

IL-6 and STAT3 are essential in the pathophysiology of multiple murine models of cachexia^17,19^. In this study, we show that interfering with eIF4A using PatA or Hipp significantly decreased IL-6 secretion and mRNA levels. Furthermore, we found significantly less STAT3 protein in the presence of PatA, Hipp and Silvestrol without a concurrent effect on STAT3 mRNA. One putative model explaining these observations is that these drugs impair the translation of the eIF4A-dependent STAT3 transcript. Consequently, the decline in total STAT3 protein levels prevents its phosphorylation and the subsequent transcriptional activation of the *IL-6* gene in the presence of IT, leading to decreased secretion of IL-6. Indeed, multiple groups have found that STAT3 activation and protein levels correlates with serum IL-6 levels in cachexia murine models as well as patients^19^. Moreover, our previous finding that STAT3 abundance and activation does not depend on extracellular IL-6 in IT-treated myotubes supports the hypothesis that the decrease in STAT3 due to inhibition of eIF4A is not caused by changes in IL-6 secretion^12^. Nevertheless, our findings do not rule out the possibility that IL-6 is dependent on eIF4A for translation and is induced independently of STAT3 in IT-treated myotubes. Indeed, a decline in IL-6 secretion in the presence of PatA and Hipp could prevent a feedback loop that maintains IL-6 mRNA levels. Therefore, more investigation into the mechanistic details of perturbing eIF4A-dependent translation in the IT-treated muscle is necessary.

The difficulty in alleviating cachexia by using monotherapies against pro-inflammatory humoral factors such as TNFα and IL-6 has been attributed to the redundancy of downstream effectors of these cytokines and the presence of multiple signaling pathways sufficient to induce atrophy. For example, STAT3 can be activated in skeletal muscle by either IL-6 or IT^12,19^. Moreover, a number of clinical trials have revealed that multimodal regimens are likely the best approach to alleviate cachexia^43^. In this study, we demonstrate that targeting eIF4A using compounds such as Hipp and PatA can decrease the amount of STAT3, a redundant downstream effector of both the IT and IL-6 signaling pathways. We also show that perturbing eIF4A-dependent translation can target multiple pro-cachectic pathways by depleting iNOS and STAT3 protein levels, both of which can induce diverse signaling changes that are sufficient for cachexia progression^7,19^. Therefore, impairing eIF4A may be effective in preclinical cachexia models because it hinders many pro-cachectic pathways as well as a common downstream effector of multiple pro-inflammatory cytokines.

Targeting eIF4A has been proposed as a potential approach for mitigating Alzheimer’s disease, cancer and viral infection^28,44,45^. This approach is  attractive for a variety of diseases because it blocks multiple pathogenic signaling pathways and proteins that may be otherwise difficult to target directly. In many of these studies, these compounds even promote the expression of proteins important in maintaining homeostasis. We suggest that drugs that inhibit eIF4A function could be useful in treating cancer cachexia. Moreover, our data provide a proof of concept that disrupting eIF4A could be a useful approach in other chronic inflammatory diseases that are driven by STAT3 and/or iNOS, such as inflammatory bowel syndrome, autoimmune disorders and other types of cancers^46,47^. Thus, targeting eIF4A is a promising treatment modality that should be seriously considered for clinical trials.

Here, we have demonstrated that perturbing eIF4A-dependent translation using Hipp recapitulates the benefits of PatA in cytokine-induced muscle wasting. Furthermore, we show that Hipp and PatA both decrease IL-6 secretion and mRNA levels in IT-treated myotubes. Finally, we reveal that targeting eIF4A with either Hipp or PatA depletes myotube STAT3 protein without a concurrent impact on STAT3 mRNA. We speculate that STAT3 is an eIF4A-dependent transcript and its decline in the presence of PatA and Hipp hinders IL-6 mRNA expression. Our findings bolster the potential of eIF4A-inhibitors in the treatment of cachexia and other pro-inflammatory diseases driven by STAT3 or iNOS.

## Materials and Methods

### Cells

As described previously^48^, C2C12 myoblasts (American Type Culture Collection) were grown in Dulbecco’s Modified Eagle Medium (DMEM, Invitrogen) with high glucose, L-glutamine, and sodium pyruvate, in addition to 20% fetal bovine serum (Sigma-Aldrich) and 1% penicillin/streptomycin antibiotics (Sigma-Aldrich). Cells were grown on tissue culture plates (Corning) with 0.1% gelatin (Sigma-Aldrich). Differentiation of the myoblasts into myotubes was triggered by switching to 2% horse serum (Gibco) and 1% penicillin/streptomycin in DMEM at 100% confluency^49^. Three days after the induction of differentiation, myotubes were incubated with or without IFNγ (100 U mL^−1^) and TNFα (20 ng mL^−1^) (IT) for 24 or 72 hours. Myotubes were incubated for 30 minutes at the beginning of IT treatment with or without Hippuristanol (100 and 200 nM), PatA (0.025 µM) and Silvestrol (12.5, 25 and 50 nM). Following this treatment, cells were washed with PBS and re-supplemented with media and IT.

### Reagents and antibodies

IFNγ and TNFα were obtained from R&D system. Myoglobin (ab77232, Abcam) iNOS (BD Pharmingen), total STAT3 (Cell Signaling), phosphotyrosine^705^-STAT3 (Cell Signaling) and α-tubulin (Developmental Hybridoma, Iowa, USA) were used.

### Immunoblotting

Western blots were performed using total protein extracts prepared in buffer containing 50 mm HEPES (pH 7.0), 150 mm NaCl, 10% glycerol, 1% Triton X-100, 10 mm sodium pyrophosphate, 100 mm NaF, 1 mm EGTA, 1.5 mm MgCl_2_, 0.1 mM sodium orthovanadate, and complete EDTA-free protease inhibitors (Roche Applied Science) as described previously^50^. Membranes were probed with antibodies against iNOS (1:5000), STAT3 (1:1000), phosphotyrosine^705^-STAT3 (1:1000) and Tubulin (1:1000). Western Blots were quantified using ImageJ.

### Immunofluorescence

Myotubes were fixed with 3% paraformaldehyde and permeabilized in 0.5% Triton X-100/PBS. Cells were then incubated with antibodies against the promyogenic marker Myoglobin (Abcam) as well as DAPI to stain the nuclei. After washing, the cells were incubated with the appropriate secondary antibody and were visualized using an inverted Zeiss Observer.Z1 (40 × oil objective) and an Axiocam MRm digital camera. Myotube widths were measured using the Axiovision software. Myotube widths were obtained by taking measurements of two points along the fiber lengths. Three fields per condition in each experiment were measured.

### Detection of NO and IL-6

Quantification of NO released was achieved using the GRIESS reagent^27^. IL-6 in the supernatant of IT-treated myotubes was discerned using the Mouse IL-6 ELISA Ready-SET-Go!® Kit (eBioscience, Inc.) as previously achieved^51^.

### Reverse Transcription PCR (RT-PCR) and Quantitative PCR (qPCR)

Total RNA was reverse transcribed with the M-MuLV Reverse Transcriptase (New England Biolabs). Resulting cDNA was diluted 1/20 and quantified using Sso Fast EvaGreen Supermix (Biorad) as described previously^48^.

Primers used include: STAT3 (Forward: 5′-GCTGCTTGGTGTATGGCTCT-3′, Reverse:5′-TATCTTGGCCCTTTGGAATG-3′) IL-6 (Forward:5′-AACGATGATGCACTTGCAGA-3′ Reverse:5′CTCTGAAGGACTCTGGCTTTG-3′), RPL32(Forward 5′‐TTC TTC CTC GGC GCT GCC TAC GA‐3′, Reverse 5′‐AAC CTT CTC CGC ACC CTG TTG TCA‐3′)

## Electronic supplementary material

Supplemental Information
