# Supplementary material for: eIF4A inhibition prevents the onset of cytokine-induced muscle wasting by blocking the STAT3 and iNOS pathways
Source: Sci Rep. 2018 May 30;8:8414. doi: 10.1038/s41598-018-26625-9 (PMC5976662; doi:10.1038/s41598-018-26625-9)
Supplement: Supplementary file 1 — Supplemental Information [file 41598_2018_26625_MOESM1_ESM.pdf]

eIF4A inhibition prevents the onset of cytokine-induced muscle wasting by blocking the STAT3 and iNOS pathways

Zvi Cramer[1], Jason Sadek[1], Gabriela Galicia Vazquez[1], Sergio Di Marco[1], Arnim Pause[1], Jerry Pelletier[1] and Imed-Eddine Gallouzi[1,2]

Supplemental Information

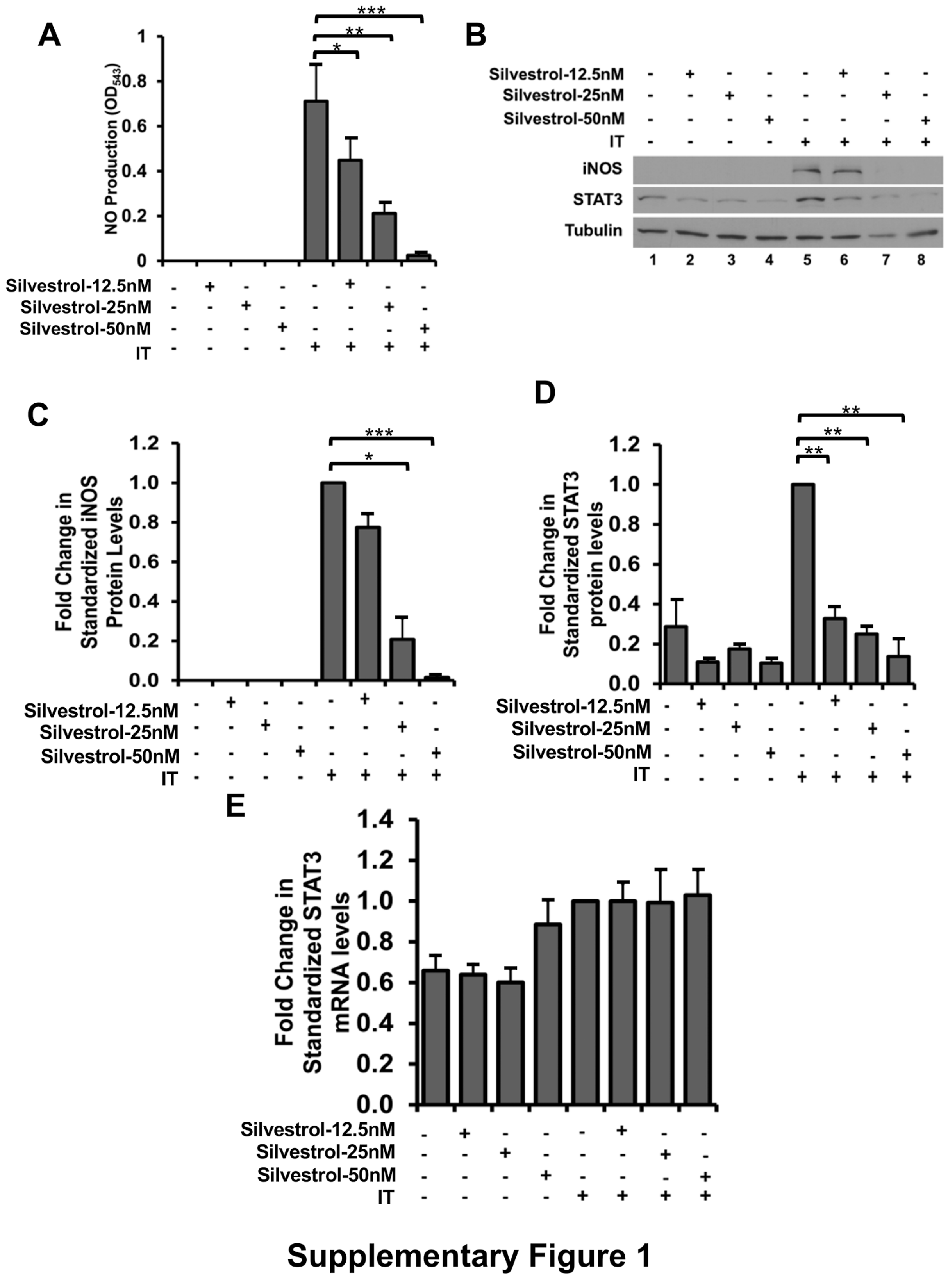

Supplementary Figure 1

__Supplementary Figure 1:__ *Silvestrol perturbs iNOS and STAT3 protein expression* **(A)** NO production was measured in myotubes treated for 24h with IT in the presence or absence of Silvestrol (12.5,25 and 50nM) using the Griess assay and plotted ± s.e.m ** p<0.01, ***p<0.001 Student's T-test (n=5). **(B)** Western blot analysis of iNOS and STAT3 protein levels in myotubes treated as described in (A). Tubulin is provided as loading control. **(C)** iNOS and **(D)** STAT3 protein levels shown in the blots in (B) were quantified, standardized to tubulin and normalized relative to IT-treated myotubes. Values were plotted ± s.e.m * p<0.05, **p<0.01, ***p<0.001 Student's T-test (n=3). **(E)** RT-qPCR analysis on RNA derived from myotubes treated as described above using primers against STAT3, standardized to the housekeeping gene RPL32 and normalized relative to IT-treated myotubes. Values were plotted ± the s.e.m. N.S. Student's T-Test (n=3).
